# Challenges and perceptions of e-learning for educational sustainability in the “new normality era”

**DOI:** 10.3389/fpsyg.2023.1104633

**Published:** 2023-01-26

**Authors:** Melinda Timea Fülöp, Teodora Odett Breaz, Ioan Dan Topor, Constantin Aurelian Ionescu, Larisa-Loredana Dragolea

**Affiliations:** ^1^Faculty of Economics and Business Administration, Babeș-Bolyai University, Cluj Napoca, Romania; ^2^Faculty of Economic Sciences, 1 Decembrie 1918 University, Alba Iulia, Romania; ^3^Institute of Multidisciplinary Research for Science and Technology, Valahia University of Targoviste, Targoviste, Romania

**Keywords:** corporate social responsibility, digitalization, technology acceptance model, universities, sustainability

## Abstract

**Background:**

All universities were faced with the challenges of e-learning when they suddenly had to switch to distance learning in March 2020 due to COVID-19 regulations. Several challenges may arise when implementing e-learning, including an insufficient budget or problems with adoption. Nevertheless, the role of digitalization is to ensure the university’s long-term sustainability. Indeed, the future of e-learning depends on future generations, which increasingly accept new technologies.

**Objectives:**

This study aimed to analyze the factors that stimulate university students’ acceptance of technology.

**Methodology:**

The study population comprised Romanian university students who took online courses during the COVID-19 pandemic. A questionnaire survey was employed to gather primary data. The surveys were distributed between January and February 2022. In particular, 1,875 questionnaires were received from students, who were the focus of this study (the results for teachers were presented in previous research). To process and interpret the data, the method of modeling with structural equations (SEM) was used. The data collected were processed using SPSS and AMOS.

**Findings:**

The results indicate that external factors do not influence perceived usefulness. Accordingly, students consider that the perceived ease of use does not influence the behavior intention to use new technologies.

**Conclusion:**

The results linked e-learning satisfaction to academic success and Romanian students who utilized e-learning during the pandemic. In addition, the results indicate that external factors do not influence perceived usefulness. Accordingly, students consider that the perceived ease of use does not influence the behavior intention to use new technologies. The results validated the basic variables of the TAM model.

**Implications:**

The study presents a series of theoretical, practical, and societal implications that can guide universities in adopting sustainable development goals.

## 1. Introduction

The fundamental changes during the health crisis imposed by COVID-19 at an academic governing level—in combination with the trend toward internationalization, the democratization of knowledge, and demographic developments—have led to new challenges in management, organization, and administration. At the same time, this health crisis has also provided entrepreneurial and institutional opportunities to rethink our organizational structures and develop and promote digitalization in the university environment (Rahman et al., 2019; Younas et al., 2022). The crisis has also encouraged university managers to think from a perspective of continuous and sustainable development (Gough and Scott, 2008; Disterheft et al., 2016). In this context, a continuous review of existing structures is needed in order to achieve added value in terms of social, ecological, and economic development (Garay and Font, 2012).

Discussions and research on corporate social responsibility in universities should not be overlooked, as they are firmly anchored in the main stakeholder groups among universities and researchers (Dobers, 2009; Johnson et al., 2018; Lu et al., 2019; Rahman et al., 2019; Torelli et al., 2020). Given its proactive role in society, the academic context is an exciting research subject. Indeed, teaching, research, and the relevant stakeholders constantly deal with issues related to various forms of corporate and social responsibility (CSR; Johnson et al., 2018; Kucharska and Kowalczyk, 2019; Saraite-Sariene et al., 2020). Curricular implementation in teaching is a prominent focus in the current discussion on CSR in the university context. There are multifaceted approaches to integrating sustainability topics into the curriculum, from single CSR or business ethics courses to holistic implementation strategies (Dobers, 2009; Jorge and Peña, 2014; Modugno and Di Carlo, 2019; Aversano et al., 2020). In the long run, a holistic approach to teaching more sustainable management can be implemented in college and anchored in the curriculum. The current state of CSR integration in undergraduate courses has been scientifically analyzed (Disterheft et al., 2016). In addition, the requirements for students for sustainability and participation have been examined. Other contributions focus on digitalization opportunities in learning and work (Pearsons, 2014; Teixeira et al., 2018; Ugwuozor, 2020). Finally, some authors have examined the UN Sustainable Development Goals (SDGs) and sustainable development framework for university management.

Our society is changing at a rapid pace due to digitalization. In particular, the Internet of Things, Big Data, robots, and Industry 4.0 are quickly becoming a reality. The new economic and living environment is increasingly characterized by flat networks and hierarchies, generating a more digital world. These developments are much more than a mere trend; they represent an evolution taking place at nearly the speed of a revolution. Therefore, some time is necessary to adjust to these changes (Knaut, 2017; Akdil et al., 2018; Scavarda et al., 2019; Ghobakhloo, 2020). Formerly stable markets (such as energy, finance, print media, and retail) are now characterized by volatility, complexity, and global cross-sector competition. In this uncertain and often contradictory world, key capabilities include coping with change and uncertainty, tolerating ambiguity, and engaging in holistic, systemic thinking. These changes increase the responsibility of people in business and society. Moreover, the speed and complexity with which business solutions must be developed require rethinking the place and type of decision-making (Barrena-Martínez et al., 2015; Aversano et al., 2020).

The new world of work will be possible only if education also considers individuals’ increased networking, self-organization, and responsibility. Therefore, methods of acquiring self-determined knowledge, as well as the innovative acquisition and processing of information, are becoming increasingly important in work and education (Dyck et al., 2019). Furthermore, in order to facilitate the free transfer of knowledge and empower people, a high level of availability is needed to openly pass on knowledge and confidently develop common problem-solving approaches (Barrena-Martínez et al., 2015; Rahman et al., 2019).

With the rise of digitalization and globalization, as well as their consequences for economic and social transformation, the future is becoming less predictable and secure. Therefore, people learn (and teach) for a world that remains largely unknown. However, it is not teaching that is crucial, but learning. Learning is a self-organized and constructivist process of acquisition. It is limited in the case of traditional, instructional knowledge transfer, which is often purely technical. However, learning is much more about transmitting the skills of transformation and reflection. In this context, economics and, therefore, managerial thinking are increasingly confronted with the major problems of the 21st century. The demand for ecological, economic, and social sustainability calls economic thinking and action into question, as well as how it is communicated in management studies at universities and colleges. Therefore, universities are responsible for reviewing their teaching concepts and constantly adapting them to new economic and social conditions (Jayakumar and Joshi, 2017; Modugno and Di Carlo, 2019; Aversano et al., 2020).

Corporate and social responsibility is an ideal topic for interdisciplinary and interdepartmental research and teaching—both within and between universities—because it crosses individual scientific departments and disciplines and can be connected to any subject (Krickhahn, 2017; Lombardi et al., 2019). With such an interdisciplinary approach to university teaching, the results of research from different scientific perspectives and their potential applications can be communicated holistically while also promoting awareness of social problems among future specialists and managers in the company. Therefore, it is essential to develop innovative and responsible skills in higher education that are appropriate to the perceived complexity of the subjects and make them accessible to society (Barrena-Martínez et al., 2015; Yang et al., 2021).

The UN’s Sustainable Development Goals (SDGs) are game-changing developments in CSR. They represent the prosaic formulation of a global vision of “the future we want.” Producing the language of the goals was an inclusive, multi-stakeholder process that began in Rio in 2012 but had its roots over three decades (with the Brundtland Report as a milestone; WCED 1987). The SDGs aim to create and amplify a wave of significant change. The end of extreme poverty, climate change, and species loss, among other changes, will fundamentally change the framework conditions of the economy. These developments will disrupt many industries and companies, forcing them to radically change their business models. However, the implementation of the SDGs can only succeed through the concept of “shared responsibility,” as there is no functioning global government institution (Fischer et al., 2015). Therefore, stakeholders in all countries are called upon to take on specific roles (Pearsons, 2014). All stakeholder groups will need to test their business models to determine whether they are ready for the future and whether continuing business as usual is a promising option (Johnson et al., 2018; Torelli et al., 2020).

For universities and companies, the SDGs represent a holistic challenge that affects all disciplines and functions: teaching, research, business, and society. As a result, priorities will change, and new content and forms of teaching and learning must be developed and implemented accordingly. A crucial question for future research remains: How can a sustainable university organization be designed to promote a holistic approach to sustainable university management—rather than only individual, often flagship, CSR projects in sub-domains (Jayakumar and Joshi, 2017; Aleixo et al., 2018)?

The activities of universities in CSR can be divided into two key parts: (1) the so-called “third mission” of the university and (2) the theme of sustainability in research, teaching, and organizational action. University departments support the university’s CSR activities in all areas and are the point of contact for questions and suggestions regarding the university and society (Barrena-Martínez et al., 2015; Lombardi et al., 2019; Modugno and Di Carlo, 2019; Rahman et al., 2019).

In recent years, the third mission of universities has been increasingly discussed and advocated. At the heart of this demand is the desire for universities to work more closely with companies and civil society to promote knowledge transfer in joint projects and to contribute to the formation of society beyond research and teaching—for example, in the areas of innovation transfer, social engagement, and further education (Barrena-Martínez et al., 2015; Aversano et al., 2020; Yang et al., 2021). Given ongoing climate change, a growing world population, and declining natural resources, sustainable development is one of the key social challenges of our time. Especially in the context of this challenge, universities are aware of their role as social models. Regardless of their freedom in research and teaching, universities uphold their students’ and employees’ commitment to sustainability (Teixeira et al., 2018; Ugwuozor, 2020). In terms of sustainable development, universities are launching selected initiatives and projects for greater sustainability and climate justice (Barrena-Martínez et al., 2015; Aleixo et al., 2018; Modugno and Di Carlo, 2019).

Universities also play a central role in digitalization and sustainability. This is where innovations are created, students learn more about sustainable and digital development, and universities have role models (Teixeira et al., 2018; Ugwuozor, 2020). In addition, a new series of lectures addresses how sustainable digitalization and digital sustainability can be promoted, researched, and put into practice (Disterheft et al., 2016; Aleixo et al., 2018). Thus, CSR has become a well-established term in recent years. For many companies, the responsible use of natural resources—including by stakeholders such as employees and suppliers—is now a matter of course (Barrena-Martínez et al., 2015; Johnson et al., 2018; Modugno and Di Carlo, 2019; Rahman et al., 2019; Torelli et al., 2020). However, what does sustainable management mean in times of digital transformation? One thing is certain: digitization is fundamentally changing the world of work. Global contact restrictions due to the COVID-19 pandemic have significantly accelerated these trends again, but digitalization also affects how we live together. Indeed, many activities are already performed by robots or intelligent machines (Scavarda et al., 2019). When referring to sustainability and its goals, universities most often mention the 10 Principles of the United Nations Global Compact (UNGC), the 17 Sustainable Development Goals, and the 2030 Learning Framework issued by the OECD (Fischer et al., 2015).

The COVID-19 pandemic has produced a swift revolution in and reimagining of higher education organizations. At the learning stage, sustainability has developed an important role over the years. It can be described as improving learning methods that can be scaled appropriately without the unreasonable exhaustion of resources or the exclusion of some populations. The 17 Sustainable Development Goals (SDGs) were designed to raise awareness of various aspects of sustainability by outlining specific targets that comprise a plan of action for a wide range of social, environmental, and technological issues. These include poverty reduction, health for all, infrastructure development, education, gender equality, and the sustainable use of oceans, energy, water, and sanitation (Crawford and Cifuentes-Faura, 2022).

Today, sustainability is no longer a mere principle of action for optimizing the use of resources in an economic sense; instead, it describes the responsible handling of our environment through technical, economic, and social implementations. Engineering sciences were and are the means of converting these resources into practice. Moreover, responsibility for the sustainable use of our environment must be recognized and accepted in teaching and research. We live and learn this principle in basic aspects of life, such as considering efficiency and sustainable construction in modern applications like smart systems. With the digitization of higher education (objective four of the UN sustainable development agenda) computer scientists and engineers play a special role in the effective use of (recorded) data for process optimization, IT, programming for resource saving, renewable energy, the energy transition, energy generation, efficient production, e-waste disposal, recycling, and other applications. Thus, new study programs must be developed in an agile, flexible, and targeted manner. In addition, courses must promote personal, ethical-social, and methodical skills more than before. Indeed, practical, technical, and interdisciplinary teaching content or partnerships with companies are more important than ever as a part of training, especially in engineering (Faura-Martínez et al., 2022). Such preparation can allow tomorrow’s engineers to better face the challenges of the digital world of work. Therefore, the successful future implementation of technology and innovation depends particularly on the successful and timely digitization of business, research, and teaching.

The purpose of the present study is to develop the influence of e-learning about CSR and the sustainability of universities, as we must be more efficient in the next years to achieve the SGDs. For universities and companies, the SDGs are a holistic challenge that affects all disciplines and functions: teaching, research, business, and society. As a result, priorities will change, and new content and forms of teaching and learning must be developed and implemented accordingly. The novelty of the study is the research area; while many studies have been conducted on the challenges and implications of e-learning and the model of acceptance of technology, we did not find any research for Romania. This represented a good opportunity and challenge to evaluate the results of e-learning after the pandemic period. We begin with a short description of sustainability and its link to social responsibility in universities to gain an overview of what we must achieve to adopt and implement the SDGs. After this short introduction, we present our hypothesis to analyze the acceptance of technology in Romanian universities. Finally, we provide a discussion, our conclusions, and the limitations of the study.

## 2. Research methodology and materials

In this section, we present the methodological conception of the paper. First, we discuss the survey tool and the methodological notations used. An analysis of the relevant papers showed that current methodical investigations have mainly been based on descriptive findings without providing definitive answers to theory-based questions. In this context, the paper’s central objective was a comprehensive behavioral analysis of the phenomenon of e-learning acceptance. The theoretical frame of reference for this discussion was provided by the technology acceptance model (TAM; Davis, 1986; Davis et al., 1989).

The psychological perspective on e-learning is sometimes neglected, as it is a special form of learning and therefore more a pedagogical topic. However, psychology is also closely related to e-learning, as psychologists are also interested in people’s learning behavior. Behavior, experience, and development play important roles in scientific psychology, and they are also influenced, among other things, by learning.

Hectic everyday life and intense stress at work and in the family are now normal for many people. The word “stress” is common in everyday language, and it is frequently used to explain a lack of time or the feeling of being overworked. Furthermore, every person experiences stress differently. In particular, the stress threshold is different for everyone: what one person perceives as a burden might seem like child’s play to another. Stress results in mental stress, which is (unfortunately) often simply accepted as normal today.

In the last two decades, disruptive technological advances have led to the collection of an unprecedented amount of data on human experience and behavior. Therefore, based on a questionnaire with 36 items, we analyze the perception of teachers and students about e-learning (acceptance of new technologies). For both questionnaires, we use a five-point Likert scale analogous to the original questions: 1 = “total disagreement” to 5 = “total agreement.” These scales were applied based on the computer-assisted web interview (CAWI) method.

A review of the relevant papers showed that current scientific research is mainly based on descriptive findings without directly addressing theory-based questions. In this context, the central objective of the present paper was a comprehensive behavioral analysis of the phenomenon of e-learning acceptance. The theoretical framework for this discussion was the TAM (Davis et al., 1989). The TAM is one of the most popular models for predicting the acceptance of technological systems. According to the model, acceptance behavior is directly determined by behavioral intention, which expresses a person’s intention to perform the behavior within a more or less precisely defined time. Behavioral intention is, in turn, determined by two factors: perceived usefulness and perceived ease of use. On the one hand, perceived benefit expresses a person’s subjective assessment of the extent to which the technological system can make a profitable contribution to the required task. On the other hand, the perceived usability of a system is described by a person.

The paper’s first research question is dedicated to the original acceptance model. It examines the extent to which the TAM is suitable for predicting e-learning acceptance. In addition to these hypotheses and findings from related research areas, the original TAM was expanded in this study to include several external factors. These factors were included because people value the benefits of the e-learning system more if they are convinced of the competence of the relevant reference person.

Specifically, the questions address perceived usefulness and perceived ease of use. The first section refers to the personal information of the students, which reflects their field and experience. The second section focuses on the level of use of e-learning. The pilot-tested questionnaires were selectively distributed among the students in order to gain a first opinion. The purpose of pilot testing these questionnaires was twofold: to observe the reliability of the questionnaire items and to confirm that the respondents could easily understand them. Care was taken to ensure that the questionnaires’ structure, language, and clarity met an acceptable standard. These surveys were distributed directly to students at three universities using a non-probability sampling approach. Data were collected from respondents who were all students. The respondents came from different institutions and different academic years, and they included male and female students. This also ensured that age was not a variable when examining usage behavior in this study.

We used quantitative data collection methods to empirically examine and highlight the factors that had a greater influence on usage behavior. A non-probability sample was adopted, which is commonly used in higher education (Acharya et al., 2013; Singh Kaurav et al., 2019). The study targeted respondents who were teaching staff or students in universities and were using university-provided e-learning system resources.

The questionnaires were distributed between January and February 2022. In total, 1,875 questionnaires were received from students, who were the aim of the present paper (the results for teachers were presented previously). In order to process and interpret the data, structural equation modeling (SEM) was used. The data collected were processed using SPSS and AMOS.

## 3. Literature review and development of research hypotheses

### 3.1. Ability to use

Ability to use (ABU) denotes the ability, skills, and self-efficacy to use the e-learning resources offered by academies/colleges and universities (Eraslan Yalcin and Kutlu, 2019). Rahmawati (2019) notes that the self-efficacy of computers has remained an influential notion in the subsequent implementation of specialized training. Al-Adwan et al. (2013) affirm that users who are self-assured in their aptitude to control an e-learning system are likely to become users of that system. Schunk et al. (2012) reveal that self-efficacy is the leading forecaster of performance and enthusiasm. Therefore, it is imperative to comprehend the computer self-sufficiency of users, their aptitude to use e-learning resources, and their predisposition to accept technology and use those resources (Gao et al., 2021; Shishakly, 2021; Fülöp et al., 2022).

*H1a*: The ABU e-learning resources positively influence perceived utility (PU).

*H1b*: The ABU e-learning resources positively influence the perceived ease of use (PEU).

### 3.2. Course content and design

When we refer to the course content and design (CCD), we refer to the accuracy, adequacy, and quality of the proposal of the materials to complement the course objectives and the educational consequences. Junus et al. (2015) emphasize that the content’s quality denotes the accuracy of the standings used, the adequacy of the resources to sustain the course’s aims, and the material’s significance. Waheed et al. (2016) argue that e-learning systems’ content must be arranged appropriately and deliver satisfactory materials. Indeed, a well-structured course increases the motivation to use the course’s materials and e-learning protocols (Arshad et al., 2021; Shishakly, 2021; Fülöp et al., 2022).

*H2a*: The CCD of the course positively influences the PU of the e-learning resource.

*H2b*: The CCD of the course positively influences the PEU of the e-learning resource.

### 3.3. Instructor contribution

The teachers’ contribution refers to the interaction between teachers and students, respectively, instructors and teachers/students. Interaction between different categories can lead to a positive relationship between learning and motivation (Arshad et al., 2021; Shishakly, 2021; Fülöp et al., 2022). Through e-learning, the instructor’s program is redefined, as well as their responsibilities and relations with students (Ejdys, 2021). According to Harandi (2015), exposed communication between the student and the teacher is a critical education factor of this platform. Moreover, Andersson et al. (2009) emphasize the importance of quality in training, as training quality is directly related to student satisfaction (Alqahtani et al., 2022).

*H3a*: The IC positively influences the PU of using e-learning resources.

*H3b*: The IC positively influences the PEU of e-learning resources.

### 3.4. Previous experience in e-learning

Previous e-learning (PE) involvement and system quality are critical determining factors of the attitude and behavioral intent to use e-learning. As revealed previously, in the present study, PE was added as an external aspect of the TAM model. Thus, we aim to examine the hypotheses linked to TAM balances and outside issues. It is therefore essential to analyze the connection between previous e-learning experiences and PU and PEU (Arshad et al., 2021; Chuenban et al., 2021; Ejdys, 2021; Shishakly, 2021; Fülöp et al., 2022). Nevertheless, various studies have reported that prior knowledge does not substantially influence user acceptance of e-learning (Hrtoňova et al., 2015). This result may also be influenced by additional issues concerning students who used e-learning throughout the pandemic without appropriate guidance. In the situation of COVID-19, Sukendro et al. (2020) reported that the PU did not meaningfully disturb students’ attitudes toward e-learning.

*H4a*: PE positively affects the PU of using e-learning resources.

*H4b*: PE positively affects the PEU of e-learning resources.

### 3.5. Quality of the e-learning system

The quality of the e-learning system (QES) refers to the quality associated with the meaning, rapidity, characteristics, and satisfaction of the earnings management scheme used in academia. Previous investigations have shown that the QES plays an important role in the PU of the e-learning system (Fathema et al., 2015; Alqahtani et al., 2022). Moreover, research has also demonstrated that the QES has significantly affected user attitudes (Mailizar et al., 2021) and the behavioral purpose of implementing the technology (Fathema et al., 2015). The outcome indicates that issues connected to the structure’s practical value - such as effortlessness of admission, the structure’s aptitude for meeting the users’ requirements, and the structure’s flexibility, are all significant and contribute to the perception of the usefulness of the e-learning system (Fülöp et al., 2022).

*H5a*: The QES positively influences the PU of using e-learning resources.

*H5b*: The QES positively influences the PEU of e-learning resources.

### 3.6. Perceived usefulness

According to Mailizar et al. (2021), for the education systems, perceived usefulness would also contain the idea of flexibility, defined as the extent to which the e-learning system’s tools and content match the students’ preferences. It includes preferred time, location, and learning style and promotes a sense of independence and self-directed learning. According to Chang et al. (2017), in e-learning, PU is defined as the degree to which employers trust that e-learning can support them in accomplishing their education and knowledge goals. An earlier investigation showed that PU has had the most critical impact on attitude (Arshad et al., 2021; Ejdys, 2021; Shishakly, 2021). In addition, PU has also had a substantial effect on behavioral intent concerning e-learning implementation (Arshad et al., 2021; Ejdys, 2021; Shishakly, 2021; Alqahtani et al., 2022; Fülöp et al., 2022). Based on previous studies, we have proposed the following hypotheses.

*H6*: PU has a significant influence on attitudes toward the use of e-learning resources.

*H7*: PU has a significant influence on personal satisfaction and development.

*H8*: PU has a positive influence on the behavioral intention to use e-learning resources.

### 3.7. Perceived ease of use

In the context of e-learning, Lin et al. (2010) define Perceived ease of use (PEU) as the degree to which users believe that using an e-learning system will be unproblematic. Earlier studies have established that PEU has meaningfully affected PU (Arshad et al., 2021; Ejdys, 2021; Shishakly, 2021). Moreover, previous studies have shown that the PEU powerfully predicts the attitude toward using e-learning (Arshad et al., 2021; Ejdys, 2021; Shishakly, 2021). The consequences of PEU on using e-learning were reported in various early studies (Salloum et al., 2019; Mailizar et al., 2021; Shishakly, 2021; Fülöp et al., 2022). As an outcome, the greater the perceived ease of using the e-learning system, the more optimistic the aim to use it and, thus, the more likely it is to be used. Furthermore, PEU should have a subsidiary outcome on the proposed use through PU in e-learning (Tawafak et al., 2020).

*H9*: The pUE has a significant influence on the attitude toward the use of e-learning resources.

*H10*: The PEU has a significant influence on personal satisfaction and development.

*H11*: The PEU positively influences the behavioral intention to use e-learning resources.

*H12*: The PEU has a positive influence on the PU.

### 3.8. Attitude toward use

According to Kaplan (1972), an attitude is a predisposition to respond to an occurrence in a favorable or unfavorable mode. Earlier investigations on e-learning acceptance have identified Attitude toward use (ATU) as a determining factor of behavioral intent concerning e-learning practices (Ejdys, 2021; Mailizar et al., 2021). Research has also shown that attitude influences behavioral intent (Chuenban et al., 2021; Ejdys, 2021; Mailizar et al., 2021; Fülöp et al., 2022; Younas et al., 2022). The association between ATU and intention emphasized in TAM suggests that attitude acts as an assessing tendency with respect to behavior. The attitude concerning e-learning reflects the extent to which an individual experiences an optimistic or damaging sensation associated with e-learning.

*H13*: There is a significant relationship between the ATU and the behavioral intention to use

### 3.9. Satisfaction and personal development

Rather than selling, supplying goods, or providing services, any professional’s primary goal is to fulfill their employers’ requirements (Ejdys, 2021; Mailizar et al., 2021; Alqahtani et al., 2022). Satisfaction and personal development (SPD) is defined as people’s perceptions of the degree to which their requirements and goals have been fully met (Ejdys, 2021), and it includes their general opinion of the computer structure (Mailizar et al., 2021). In some studies, SPD has demonstrated favorable results for e-learning facilities (Ejdys, 2021; Mailizar et al., 2021; Younas et al., 2022). It has also shown substantially optimistic results for ATU (Ejdys, 2021). According to Bolliger and Wasilik (2009), there is a link between SPD and performance. Consequently, educational institutions must do their utmost to meet the needs of students, as this may increase their academic performance. Moreover, it has been shown that satisfaction with e-learning platforms is also influenced by PU and PEU (Ejdys, 2021; Fülöp et al., 2022). The association among these items is grounded on the idea that employers will not be fulfilled if they believe that a specific structure will not help advance their performance or is challenging to use.

*H14*: There is a significant positive relationship between SPD and behavioral intention to use.

### 3.10. Behavioral intent to use

Intention, which is the key dependent variable recognized in TAM-based investigations, is defined as the probability that an employer will use a computer system. Purpose plays a critical role in the use of new technology (Davis et al., 1989). Behavioral intent to use (BIU) can also be considered as an attitude (Ejdys, 2021). In the acceptance field, researchers have investigated the association between the BIU and its actual use in e-learning (Ejdys, 2021; Mailizar et al., 2021). Thus, BIU is another factor in the adoption of technology. Davis et al. (1989) postulates that ease of use is associated with the willingness to apply the technology. Relatedly, Sun et al. (2008) relate the constructive approaches concerning technology and the perceived BIU for e-learning. According to Abdel-Wahab (2008), e-learning is inclined by reliable admission to e-learning. Furthermore, BIU facilitates the association among real users, PEU, and PU. These terms become helpful when analyzing the prediction of appropriate behavior, which specifies employees’ willingness to take voluntary action. The willingness of employers to use new information technology is recognized as the purpose of using new information technology. Based on this research, we propose the following hypotheses (Fülöp et al., 2022):

*H15*: BIU e-learning resources have a positive effect on academic performance.

*H16*: BIU significantly influences the actual use of e-learning resources.

### 3.11. Actual use

Many challenges disturb the practical usage of e-learning. Both Erarslan and Seker (2021) and Almaiah et al. (2020) argue that numerous limitations affect the efficiency of e-learning use. These challenges can be attributed to the organizational, technical, application, social, cultural, industrial, and specific course-related challenges. Moreover, the model of accepting technology is still being questioned. The analysis showed that most previous studies on actual use (AU) and the adoption of the e-learning platform were conducted using quantitative methodologies. Therefore, the AU variable has been added to the model. However, as the utilization rate of learning management systems differs between the two groups, the reliability of the AU items is low (Fülöp et al., 2022).

### 3.12. Academic performance

The TAM model has been adapted to assess the support and improvement of academic performance. Therefore, TAM is used as a framework for understanding the effectiveness of learning improvement through adapted technologies, which use many factors to influence their decision (Tawafak et al., 2021). The measures used to represent academic performance (AP) were adapted from research conducted by Islam (2013, 2016). AP assesses the student’s e-learning situation, while personality-education aptitude is an assessment of one’s personality-education ability. This method exposes the broad and accurate tendencies demonstrated by e-learning studies. Given the theory of customer performance, fulfillment is measured by the client’s reply concerning their self-actualization, as well as their decision about the product or facility. Satisfaction also includes meeting one’s desired performance (Tawafak et al., 2021; Fülöp et al., 2022); in the academic context, this means improving academic performance. In this sense, we can say that satisfaction is forecast by perceived usefulness, the superiority of facilities, and evidence. It is reasonable to expect that using an e-learning structure provides such support to students. Therefore, we claim that the support offered by the e-learning structure will ultimately influence the students’ perceived AP.

**Figure 1:**
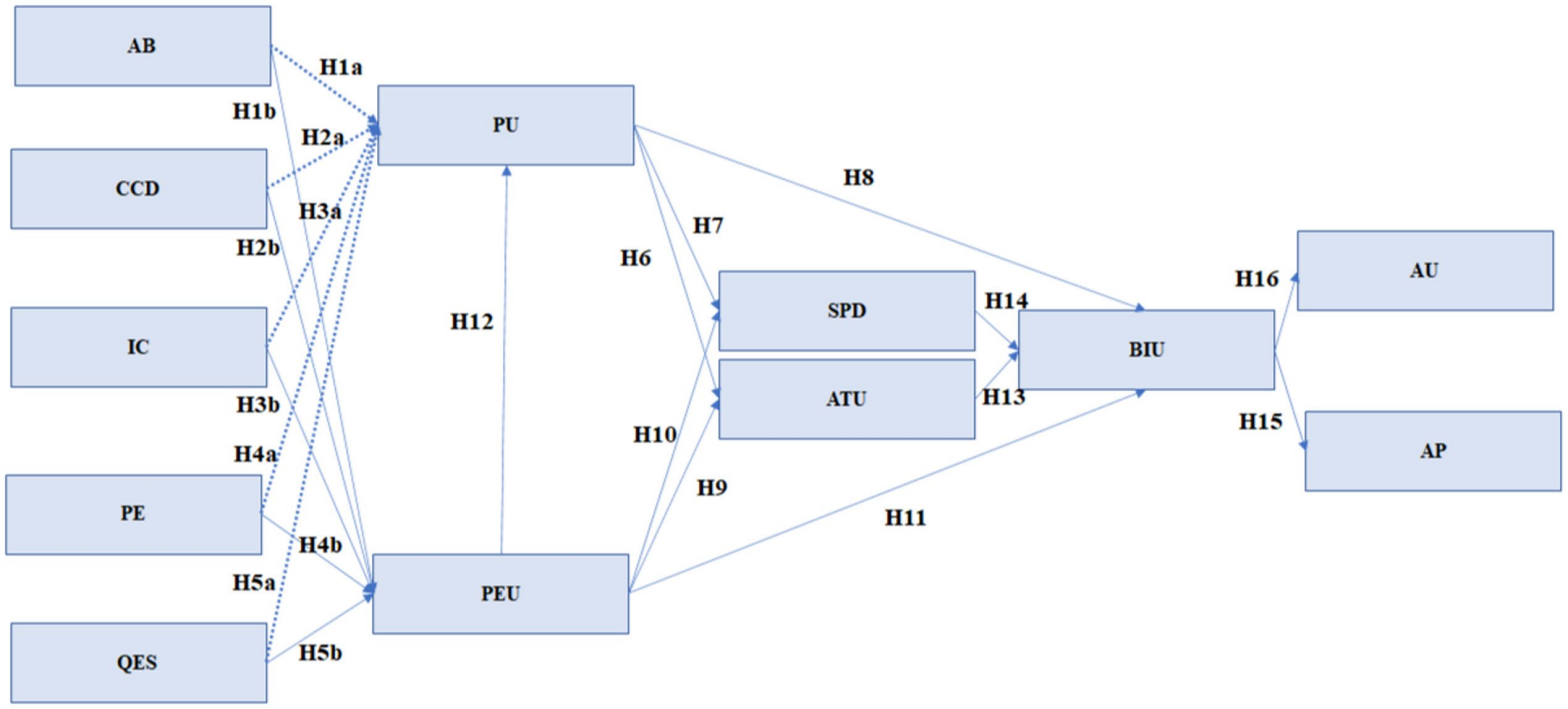
Hypothesis development.

Based on the specialized literature, we present an analysis model regarding the acceptance of technology by students (Figure 1).

Following established research hypotheses and data collection, we analyzed and interpreted this model with the help of statistical software.

## 4. Results and discussion

When using new information and communication technologies, it is also necessary to explore the possibilities and limitations of digital literacy. Our studies provide interesting starting points for further considerations on this topic. Questions have been debated about the influence of e-learning on the skills possessed by students and teachers, but also about changing the approach to courses and seminars. From the point of view of knowledge management, there are questions concerning how to deal with complex and networked information, including the didactic question of the possible perspectives of multimedia teaching. Findings from the student case study show that, following the increasing digitalization of learning worlds, structural change is underway.

The characteristics of the sample are presented in Table 1, which shows that 67.04% of those sampled were undergraduate students, 32.64% were master’s students, and 0.32% were doctoral students. We also note that 58.99% of respondents fall into the 18–25 age group, and a percentage of 6.77% are over 45 years old. All 1,875 respondents were students at a public or private university.

**Table 1:** Demographic results regarding students involved in the study.

	No. respondents	Percentage
Category of students
Bachelor	1,257	67.04%
Master	612	32.64%
PhD	6	0.32%
Total	1,875	100%
Age group
18–25 year	1,106	58.99%
25–35 year	362	19.31%
35–45 year	280	14.93%
Over 45 year	127	6.77%
Total	1,875	100%
Environment
Urban	1,236	65.92%
Rural	639	34.08%
Total	1,875	100%
The type of university
Public	1,783	95.09%
Private	92	4.91%
Total	1,875	100%

While using new information and communication technologies, it is necessary to explore the possibilities and limitations of digital literacy, for which our studies provide interesting starting points for further consideration. The questions referred to the influence of e-learning on the students’ and teachers’ skills, as well as on how courses and seminars are handled. In terms of knowledge management, the question relates to dealing with complex information in the network, from didactics to the issue of possible perspectives in multimedia teaching. The findings of the case study of students show that a structural change is underway due to the increasing digitalization of learning worlds. In order to provide an overview of the results obtained, we present the results of the empirical study below.

Before we examined the hypotheses, we applied the test of validity and reliability of the variables, following best practices. Validity can be measured by analyzing the main elements with Kaiser Varimax rotation (Kaiser, 1970, 1974; Hair et al., 1999). As the Kaiser-Meyer-Olkin & Bartlett test results show, our elements fall within the recommended range between 0.8 and 1, with a value of 0.946 and a significance of 0.000, indicating adequate sampling. We also performed a reliability test. The results confirm that the sample is adequate, with a value for Cronbach’s alpha of 0.923 and Cronbach’s alpha based on standardized items of 0.929. The factor loading is the correlation coefficient for the variable and the factor (Table 2); it displays the variance elucidated by the variable for that factor. In structural equations modeling (SEM), as a general guideline, a factor loading of 0.7 or higher means that the factor extracts sufficient variance from that variable. As our results are above 0.7, we can consider them adequate.

**Table 2:** Factor loadings and items’ reliability.

Elements	Factor loading internal	Composite factor reliability ≥ 0.70	Average variance extracted ≥ 0.50
ABU	0.92	0.912	0.84
	0.91		
	0.91		
CCD	0.89	0.913	0.83
	0.92		
	0.91		
IC	0.88	0.901	0.80
	0.91		
	0.92		
PE	0.81	0.912	0.79
	0.90		
	0.93		
QES	0.87	0.921	0.80
	0.92		
	0.94		
PU	0.89	0.907	0.81
	0.86		
	0.91		
PEU	0.91	0.817	0.87
	0.87		
	0.89		
SPD	0.88	0.877	0.88
	0.92		
	0.93		
ATU	0.91	0.902	0.83
	0.88		
	0.92		
BIU	0.89	0.899	0.82
	0.90		
	0.94		
AU	0.82	0.905	0.89
	0.87		
	0.85		
AP	0.91	0.897	0.86
	0.86		
	0.87		

The correlation matrix of the data set is shown in Table 3. Correlations greater than 0.3 were statistically significant at 0.01. In our correlation matrix, most correlations between items were significant at 0.01, with values greater than or equal to 0.3.

**Table 3:** Correlation matrix.

	1	2	3	4	5	6	7	8	9	10	11	12
ABU	1											
CCD	0.516	1										
IC	0.487	0.685	1									
PE	0.344	0.343	0.303	1								
QES	0.473	0.595	0.644	0.375	1							
PU	0.535	0.758	0.566	0.322	0.513	1						
PEU	0.611	0.609	0.519	0.276	0.496	0.685	1					
SPD	0.497	0.741	0.529	0.358	0.483	0.744	0.594	1				
ATU	0.553	0.697	0.545	0.326	0.521	0.733	0.652	0.734	1			
BIU	0.494	0.753	0.531	0.361	0.473	0.762	0.605	0.827	0.755	1		
AU	0.371	0.403	0.387	0.348	0.445	0.361	0.373	0.361	0.425	0.374	1	
AP	0.445	0.653	0.549	0.337	0.601	0.607	0.515	0.594	0.606	0.619	0.417	1

We completed the primary analysis of the reliability of the measurement scale of our model by applying Cronbach’s alpha, the most generally used indicator for this category of analysis. This coefficient covers values between 0 and 1. Table 4 shows the Cronbach’s alpha coefficient with values above 0.900, indicating that the apparatus can be reliable and internally consistent.

**Table 4:** Reliability of elements based on Cronbach’s alpha.

Elements	Mean	Cronbach’s alpha
ABU	4.50	0.919
CCD	4.28	0.910
IC	4.41	0.916
PE	3.41	0.936
QES	4.55	0.917
PU	4.30	0.911
PEU	4.40	0.915
SPD	4.26	0.911
ATU	4.50	0.911
BIU	4.35	0.910
AU	4.41	0.924
AP	4.35	0.915

After the satisfactory results concerning reliability, correlations, and validation, we continued with the analyzes of the model’s degree of fit and adequacy. To assess the model’s fit, we tracked the current standards for the use of several adjustment indicators (Hu and Bentler, 1995; Hu et al., 1999). We used several indices. First, we employed chi-square, for which a value less than 3 is considered a good fit in our case with a value of 2,943 (Sanchez and Hueros, 2010; Tarhini et al., 2014; Ros et al., 2015). We also used GFI (goodness-matching), which varies from 0 (bad fit) to 1 (perfect fit). Chang & Tung, 2007 recommend a value ≥0.8, according to which our results (0.911) are also satisfactory. The AGFI (adjusted goodness of fit) of 0.848 is within the acceptable value: greater than or equal to 0.8. Our sample NFI (standard fit index), with a value of 0.901, is within the recommended range of 0 and 1 (Ros et al., 2015). The Comparative Match Index (CFI), with a value of 0.934, fluctuates between 0 and 1 according to the literature (Byrne, 2010; Sanchez and Hueros, 2010; Tarhini et al., 2014; Ros et al., 2015). With a value of 0.061, RMSEA (root mean square error of approximation) fits well between 0.05 and 0.08 (Sanchez and Hueros, 2010; Tarhini et al., 2014).

**Figure 2:**
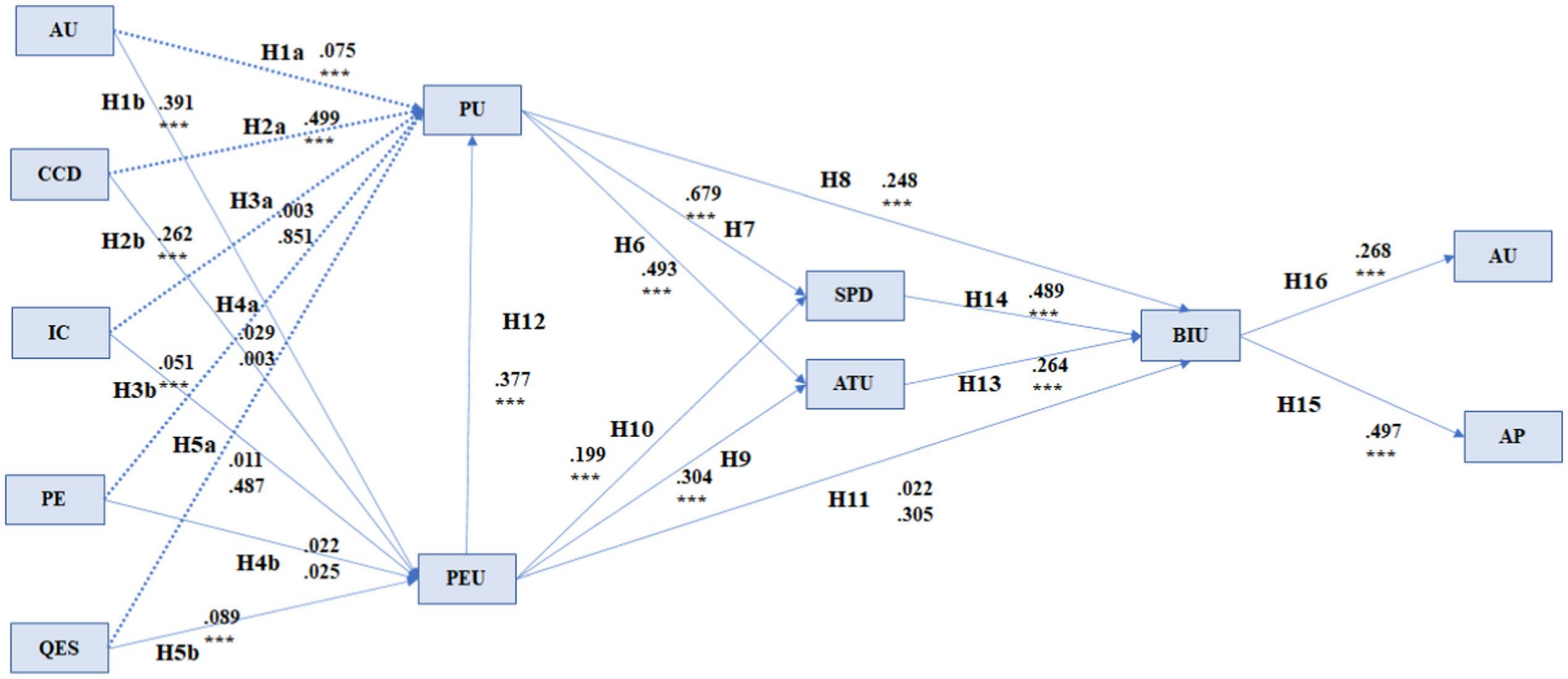
Hypothesis validation. ***p = 0.001.

As all preliminary tests were within an acceptable range, we moved on to the last stage: analyzing the estimates based on path analysis (path coefficient) to validate or reject the assumptions. The results of the analysis are presented in Table 5. As observed, of the 21 hypotheses, five were rejected because they had a *p*-value higher than 0.001, indicating no or insignificant influence between the variables (Figure 2; Table 5).

**Table 5:** Hypothesis validation.

Hypothesis	Path coefficient	*p*	Validation
H1a	0.075	0.001	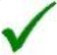
H1b	0.391	0.001	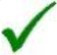
H2a	0.499	0.001	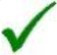
H2b	0.262	0.001	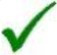
H3a	0.003	0.851	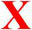
H3b	0.051	0.001	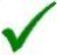
H4a	0.029	0.003	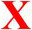
H4b	0.022	0.025	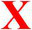
H5a	0.011	0.487	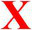
H5b	0.089	0.001	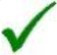
H6	0.493	0.001	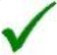
H7	0.679	0.001	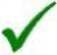
H8	0.248	0.001	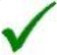
H9	0.304	0.001	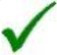
H10	0.199	0.001	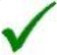
H11	0.022	0.305	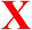
H12	0.377	0.001	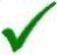
H13	0.264	0.001	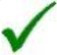
H14	0.489	0.001	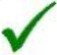
H15	0.497	0.001	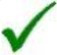
H16	0.268	0.001	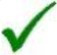

Our results align with other field studies (Šumak et al., 2011; Mohammadi, 2015; Doleck et al., 2018; Eraslan Yalcin and Kutlu, 2019; Al Kurdi et al., 2020; Shishakly, 2021).

Students’ use of skills is a necessary process in the context of the ability to learn in an online e-learning environment. In addition to successfully implementing professional knowledge, personal mastery includes coping. In this context, one’s personality is understood as a learning task that involves learning from mistakes and inventing new things. Values, visions, personal goals, and ideas play an important role here. Moreover, self-control allows students from different backgrounds to improve educational processes through e-learning platforms. The learning content presented should equally appeal to students with different media skills so that even students with little media experience can quickly gain a sense of accomplishment—for example, through help pages for relevant learning environments. However, students are not only heterogeneous in terms of media skills but also unique in their personalities (Mohammadi, 2015; Eraslan Yalcin and Kutlu, 2019; Pimdee, 2020; Shishakly, 2021).

The possible uses of e-learning in everyday university life begin with synchronous and asynchronous work. For example, the availability of content on the platform allows students to catch up and recover if they missed something or are sick. As a result, students most clearly experience a change in their learning environment. The student also stands to benefit from blended learning when it comes to learning and understanding: the clarity of the content makes it possible to prepare and present it following a more individualized approach (Šumak et al., 2011; Shishakly, 2021).

According to the results of this comprehensive study, setting clear learning goals and striving for them actively and in a focused manner contribute to student success. Being present often enough to attend courses and seminars is also crucial. The technological factors of e-learning, such as digital learning environments, only lag when it comes to increasing student performance (Mohammadi, 2015; Al Kurdi et al., 2020). Lively, face-to-face, clearly structured teaching still appears to be the best way to study and learn successfully. Even if the courses and seminars are not designed to be as varied, there should be opportunities to talk to classmates, ask the teacher questions, and develop new ideas in conversations. The experiences of the students surveyed with the learning platform were ambivalent with regard to these features.

On the one hand, e-learning has been seen as a valuable tool for effective learning and administration; on the other, it is not considered user-friendly because it is too complicated to use. Studies have shown that familiar things can be processed—and thus learned—more easily and quickly. Consequently, it is essential to support the use of digital space, such as games, social media, video, and audio. In our free time, we are accustomed to accessing content from the internet *via* our smartphones. Therefore, the content provided should also meet these requirements within the university. Students must be able to access the content both through desktop computers and through tablets or smartphones. Thus, the content should be optimized accordingly in terms of content and form. Such optimization may mean, for example, that some images, texts, or task types are much easier to use on a tablet or smartphone (Mohammadi, 2015; Eraslan Yalcin and Kutlu, 2019; Shishakly, 2021).

It is essential not only to present learning material with the highest quality and an attractive design but also to prepare it to gain students’ attention and interest, which is a prerequisite for storing learning content according to methodical and didactic principles. Methodology and didactics help students both absorb and transfer the learning content. In addition to perceived ease of use, a decisive factor for the acceptance of the use of new media by students who use the system as a learning environment is the media’s lack of competitiveness compared with established learning platforms that are specialized for particular subjects (Šumak et al., 2011). A wide range of platforms for individual subjects offer teaching and learning materials, meaning that teachers do not have to develop the materials themselves. When a person decides whether the newly introduced information system is proper (perceived usefulness), the results’ quality plays a crucial role (Mohammadi, 2015; Al Kurdi et al., 2020).

The perceived benefit also depends on the extent to which the technological systems are (or were already) present in daily activity, as well as how relevant this system is for the person’s immediate context. Acceptance of behavior is determined by perceived benefit and perceived ease of individual use. The participants’ perceived benefits were directly related to the efficient management of time; they also considered that the use of e-learning tools and methods improves the results and increases the quality of the learning process (Doleck et al., 2018; Al Kurdi et al., 2020).

Perceived ease of use also plays a vital role in accepting e-learning. Specifically, it must include the following aspects: the highest quality, an attractive design, a well-thought-out user guide, methodology and teaching, adequate consideration of different media skills and personalities, appropriately adapted usage habits, and an effective manner of providing the learning. Many of the relevant facts may be familiar from everyday life. Moreover, if we need help with a problem, we use a search engine (Mohammadi, 2015; Shishakly, 2021).

The literature provides some clues about how acceptance and use can be achieved. One sensitive approach is to use concrete e-learning systems to identify the factors that led to a high level of acceptance and intensive use later—that is, after the students used them. It can also be assumed that the didactic quality of the e-learning platform has an influence (Šumak et al., 2011; Doleck et al., 2018). In many cases, the benefits of using e-learning cannot consistently be implemented to the satisfaction of teachers and students; such platforms’ use has not always been appropriate and, in some cases, is rejected. Students consider that e-learning gives them flexibility, and they like the system; nevertheless, this comes at the expense of the creativity they could show in courses and seminars, as shown in previous research (Al Kurdi et al., 2020).

On the one hand, in principle, e-learning is appropriate if well-founded theoretical knowledge is transmitted with a large proportion of text, video content, or sound sequences that require a longer duration of attention. On the other hand, mobile learning is particularly suitable for consolidating what has been learned using small learning bits. The use of e-learning in the classroom is also closely linked to the issue of added value. Given the new technical possibilities, this added value is evident in new digital systems’ unique capabilities. Finally, we note that most students use the e-learning system frequently; they used it not only during the pandemic period but also before the e-learning system was used in most courses and seminars (Doleck et al., 2018; Shishakly, 2021).

The impact of e-learning is assessed by determining whether students understand what has been offered or taught to them. The literature has demonstrated that, compared to traditional approaches, e-learning reduces students’ ability to understand what is being taught or delivered. As the results show, our students consider that the e-learning system allows proactive communication with the teaching cards; moreover, they experienced a development in their ICT skills through the system (Šumak et al., 2011, Doleck et al., 2018). However, as we do not consider that the e-learning system contributes significantly to the increase in assessment marks, we note that the system does not offer a clear advantage in knowledge assessment. Indeed, less than half of students observed an increase in assessment through e-learning (Al Kurdi et al., 2020).

The sustainability of higher education is essential for national, regional, and global development. Rapid increases in the capacity and capability of information technology and communication channels have accelerated the transmission and impact of information. However, the nature and extent of their benefits vary widely across the globe, primarily due to differences in existing infrastructure and the availability of skilled labor. Nevertheless, as a center for the generation and dissemination of knowledge, higher education is ethically bound to develop professionals with the knowledge, skills, and competencies necessary to address the evolving dynamics facing individuals, organizations, and society.

Education is key to sustainably shaping the future. In particular, management theories have the power to change the real world. For example, in the late 90s, the shareholder value approach or agency theory influenced the management of companies and universities. Today, there is more and more talk of the 2030 Agenda for Sustainable Development issued by the United Nations (n.d.), as well as the 2030 learning framework issued by the OECD. In order to support the implementation of the 2030 Agenda’s sustainable development objectives, universities must change their approach to organization and management in order to achieve objective 4, which aims at high-quality education. A national strategy would be welcome in order to implement the basic objective of quality education. However, no university should wait for government action, as there are actions that universities can implement autonomously.

Universities are of particular importance when it comes to implementing the objectives. Indeed, universities are where knowledge, innovations, and solutions are created and developed, as well as where future decision-makers are trained and empowered to act. Universities transmit people and skills to society, influencing social discourse and debate.

More and more universities are becoming aware of their social responsibility, their role as a model, and the opportunities associated with that role. Such universities anchor sustainability in their mission statement, establish sustainability advisory boards, and create national university networks for sustainability. In addition, more and more universities are not only addressing the topic of sustainability reporting in teaching and research but are also using such reporting to showcase their institution’s contribution to sustainability.

Given all the roles that universities play for regions and countries, they are in a unique position to influence the culture in the areas in which they operate. Universities can become cultural change agents of sustainable development by following two paths. The first path leads to the development of sustainability through internal changes in the university culture. This approach aims to recognize sustainability as a goal and to ensure the appropriate quality of operations, thereby influencing university stakeholders by setting an example and becoming a role model by adhering to the principles of sustainability. The second path is based on the effects that higher education can achieve on university stakeholders through its three major functions: teaching, research, and wider societal engagement.

Further investigation into the use of e-learning system resources is needed. This can be attributed to both the continuous and rapid increase in dependence on technology and the continuous developments in the education sector worldwide. In the present context, the use of TAM supports additional factors included in the model. Furthermore, this study adds value to the existing literature by investigating the factors that influence the use of e-learning resources by student teachers; such resources are provided by their universities to enhance their learning processes anytime and anywhere. The results of the study facilitated a deeper understanding of external factors among faculty and students and provided information for university managers, designers, system developers, and related professionals. Although technology is used to an effective degree in Romania and its higher education institutions, more attention must be paid to factors that have a relevant role in facilitating the use of e-learning systems by students. Such a focus could further improve performance and efficiency between students and teaching staff, respectively, to achieve sustainability objectives. Finally, the results of the study provide a deeper understanding of the dominant and significant factors that influence the use of learning resources by teaching staff and students in Romanian universities.

Second, the study provides universal analyzes of the factors and challenges that influence faculty and student acceptance of e-learning use. These include students’ ability to use technology, management challenges, implementation, cultural factors, self-efficacy, peer influence, course design, instructor input, and financial constraints. These factors have been classified in a variety of ways depending on the perspective of the study.

Third, at the managerial level, the results of this study allow us to make recommendations for managers, developers, designers, and decision-makers in universities seeking to promote the use of e-learning by ensuring system quality and modifying functionality. This can lead to higher-quality course content and improved use of e-learning resources among students and teachers, respectively, thereby achieving sustainability goals.

Based on the findings of this study, there is a notable effect of use intention on e-learning, as it correlates with teachers’ usage behavior, perceived ease of use, and perceived usefulness. Therefore, system developers, designers, and universities should consider the accessibility, functionality, interactivity, audio, and video facilities of the systems to ensure students’ engagement and usage intention in a more effective way.

Finally, the latest technology will continue to be used in Romania, while former e-learning systems will become redundant. Therefore, it is essential to continue ongoing research on this technology as well as to increase the understanding of the most relevant factors that support the use of e-learning in higher education. As the factors influencing the use of e-learning by teachers and students and technology work in tandem, they are susceptible to change alongside the priorities and adaptability of changing technologies. The adaptability of these technologies will work alongside e-learning in universities, and the dominant factors will change.

## 5. Conclusion and implications

### 5.1. Theoretical implications

Education is the key to sustainably shaping the future. In particular, management theories have the power to change the real world, just as the approach of shareholder value or agency theory in the late 1990s influenced the management of companies and universities. In the OECD’s 2030 learning framework to support the implementation of the sustainable development goals of the 2030 agenda, universities must change their organization and management to achieve the fourth goal of quality education. A national strategy would be welcome to implement the fundamental objective of quality education. However, no university should wait for government action, as there are actions that universities can implement autonomously.

Universities are of particular importance when it comes to implementing goals. Indeed, universities are where knowledge, innovations, and solutions are created and developed, as well as where future decision-makers are trained and empowered to act. Universities transmit people and skills to society, influencing social discourse and debate.

More and more universities are becoming aware of their social responsibility, their role as a model, and the opportunities associated with that role. Such universities anchor sustainability in their mission statement, establish sustainability advisory boards, and create national university networks for sustainability. In addition, more and more universities are not only addressing the topic of sustainability reporting in teaching and research but are also using such reporting to showcase their institution’s contribution to sustainability.

The discussion on improving teaching quality has already put considerable pressure on teachers to develop as people and look for a new position concerning their attitudes and teaching techniques. Education for sustainable development has increased this pressure by aiming to develop the whole teaching-learning process into a process of equal communication in which all those involved learn how to shape the transformation of the economy and society.

Various trends and developments in recent years can be identified in and derived from digitalization and digital transformation. Both digital offerings and global digital markets are evolving at an unprecedented pace, creating new trends and opportunities for capitalization. In particular, the construction of a virtual world must be seen as a milestone in the age of digitalization. This is where the opportunities for universities lie—in increasing competition with new and potentially huge needs. In this context, universities must become more mobile and connected to new digital technologies in order to meet the demand for educational offerings anywhere and anytime.

In short, digital media presents innovative opportunities, especially in opening new markets and capitalizing opportunities. For today’s universities and companies, it is vital to develop concrete implementation and strategy models, create appropriate jobs, develop new job profiles, and promote future specialists.

### 5.2. Practical implications

Sustainable management models are based on the principles of sustainability (sustainable development) and corporate responsibility in combination with various management concepts. Sustainable management can thus be described as an entrepreneurial practice that applies sustainability concepts to create added value for companies, society, and the environment. Given the ongoing transition from traditional to sustainable management, a future orientation toward management studies is essential, as today’s students are tomorrow’s decision-makers.

The management approaches and perspectives of the last century initially met with great success in a time of supposedly unlimited resources. However, this changed when global conditions began to change massively (if not earlier). Limited resources, demographic changes, and especially the financial crisis have shown that profits cannot be made at the expense of third parties or the environment in the long term. This has resulted in completely new challenges for both companies and universities. In particular, today’s students—who are tomorrow’s managers—must place special emphasis on the social, environmental, and economic dimensions. Therefore, representatives of management leadership are demanding that business models that have often been successful to date be adapted to current conditions, such as incorporating CSR in a sustainable management approach. However, we must not forget that the requirements of sustainable management have real consequences for universities as well, which must rethink their own actions and revise existing programs. As such, universities benefit from support from the United Nations under the Principles for Education for Responsible Management and the Sustainable Development Goals, which are respected by more and more universities. The new management paradigm associated with the SDGs also means that all individual scientific disciplines include the subject of responsibility and sustainability in their specialist discourse. Existing higher education strategies and educational discourses will be rethought and organized in this context. If universities manage to address both entrepreneurial and social added value in their education, they will create a sustainable, future-ready educational paradigm.

The present study showed that the acceptance of e-learning can be explained as a function of personal determinants (e-learning). This finding allows for the development of promising intervention measures for e-learning. If it can influence one or more predictors, the acceptance will also change.

The study has several limitations. The first is the number of respondents. Second, we only analyze the acceptance of technology from the student’s point of view. A possible further analysis can also involve teachers or a comparative examination of the technology acceptance from both categories.

### 5.3. Societal implications

Digitization will significantly shorten innovation cycles. While digital tools and new technologies are creating new fields of work, they are also making the working world of engineers more complex and confusing. Precisely due to this new complexity, students must learn flexibly and methodically during their studies. Therefore, it is important that students broaden their knowledge in an engineering discipline with a solid foundation in digital disciplines.

In order to prepare for the future world of work, engineering courses must become even more practical. Universities are increasingly reacting to this need by establishing practice-oriented learning factories in cooperation with companies or by providing practical projects as a guide for engineering studies. The focus must be on enabling didactics and learning based on concrete practical problems.

Sustainability depends on independent, well-founded research. On the one hand, this research must expand the understanding of the causes, effects, and interdependencies of global phenomena such as climate change. On the other hand, it must develop innovative technologies and strategies for success. The change toward a sustainable society is a transversal problem that affects various specialist fields. Therefore, it requires more interdisciplinary and transdisciplinary research that takes a holistic view, sets practice-oriented solutions and priorities, and supports the transfer of solutions into practice. Transparency in the financing of research projects and the promotion of young researchers is also important. The direct flow of knowledge from research to teaching is of added value to students and should be used accordingly.

Increasing the level of integration is currently an important political objective for European research. Indeed, research integration provides the opportunity to find new solutions to existing and future challenges, and integration across disciplines and between research and practice is increasingly recognized as essential to address complex problems more effectively. In this context, more and more diverse parts of universities have become involved in research aimed at contributing to different aspects of sustainability. Moreover, dedicated academic departments have been formed in many universities to undertake more integrative studies. Given the results of the present study, we recommend that universities gradually try to move from disciplinary research to interdisciplinary and transdisciplinary research.

## Data availability statement

The original contributions presented in the study are included in the article/supplementary material, further inquiries can be directed to the corresponding author.

## Author contributions

MF, IT, and CI: conceptualization. TB: methodology and funding acquisition. MF: software and data curation. IT: validation and visualization. L-LD: investigation. IT: resources. L-LD and TB: writing–original draft preparation. CI and MF: writing – review and editing and supervision. MF: project administration. All authors critically revised the manuscript and gave their final approval of the manuscript submitted for publication.

## Conflict of interest

The authors declare that the research was conducted in the absence of any commercial or financial relationships that could be construed as a potential conflict of interest.

## Publisher’s note

All claims expressed in this article are solely those of the authors and do not necessarily represent those of their affiliated organizations, or those of the publisher, the editors and the reviewers. Any product that may be evaluated in this article, or claim that may be made by its manufacturer, is not guaranteed or endorsed by the publisher.
